# Controlled Synthesis and Formation Mechanism of Uniformly Sized Spherical CeO_2_ Nanoparticles

**DOI:** 10.3390/ma19010211

**Published:** 2026-01-05

**Authors:** Jiayue Xie, Kai Feng, Rui Ye, Maokui Wang, Yunci Wang, Xing Fan, Renlong Liu

**Affiliations:** 1 College of Chemistry and Chemical Engineering, Chongqing University, Chongqing 401331, China; 15271029899@163.com (J.X.); 17264363577@163.com (K.F.); 15971061997@163.com (R.Y.); 202318131158@stu.cqu.edu.cn (M.W.); 13339827815@163.com (Y.W.); 13581427088@163.com (X.F.); 2 Hubei Sinophorus Electronic Materials Co., Ltd., Yichang 443007, China; 3 State Key Laboratory of Coal Mine Disaster Dynamics and Control, Chongqing University, Chongqing 400044, China

**Keywords:** CeO_2_ nanoparticles, molten salt method, spherical morphology, synthesis mechanism, chemical mechanical polishing (CMP)

## Abstract

As the core abrasive in chemical mechanical polishing (CMP) processes, the morphology, size uniformity, and chemical reactivity of CeO_2_ nanoparticles (NPs) are crucial factors determining the surface precision and yield of devices. In this work, a KNO_3_–LiNO_3_ eutectic molten salt was used as the reaction medium. By systematically adjusting key processing parameters (such as the type of cerium source, the species and dosage of surfactants, and calcination conditions), the regulatory effects of these factors on particle growth mechanisms were clarified. This adjustment enabled the controlled synthesis of spherical CeO_2_ NPs with customized morphology, particle size, and surface defect states. The multi-stage reaction process of the precursor during calcination was identified by applying thermal analysis techniques, including TG-DSC and TG-FTIR. This process includes dehydration, ion exchange, and thermal decomposition. Microstructural analysis shows that the type and dosage of the cerium source and template agent significantly affect the uniformity of particle size and spherical morphology. Moreover, by using an optimized process with a heating rate of 6 °C/min and maintaining at 400 °C for 3 h, spherical CeO_2_ NPs with an average particle size of 60 nm, uniform size distribution, and high sphericity were successfully synthesized via a single-step calcination process. Based on these findings, a further proposal was put forward regarding a crystal growth mechanism mediated by micelle-directed assembly and oriented attachment. This method only requires a single calcination step, has mild reaction conditions, and involves a simple process without the need for specialized equipment—features that show great potential for scalable production. It provides both a theoretical basis and experimental support for the controlled preparation of high-performance CeO_2_ abrasives.

## 1. Introduction

Cerium dioxide (CeO_2_) is a prominent rare earth oxide with a series of advantageous physicochemical properties. These include low cost, high oxygen storage capacity, low toxicity, reversible Ce^3+^/Ce^4+^ redox transitions, and abundant oxygen vacancies (Vo). Due to these properties, it is widely used in multiple fields such as chemical mechanical polishing (CMP) [1], solar cells [2,3], solid oxide fuel cells [4], biosensing [5], pollution treatment and environmental protection [6], and industrial catalysis [7]. Furthermore, in the CMP processing of silica, Ce^3+^ sites on the surface of CeO_2_ abrasives chemically interact with SiO_2_, forming Ce–O–Si bonds. This significantly enhances the material removal rate [8]. Overall, due to its unique affinity for SiO_2_, appropriate hardness (Mohs hardness of ~7), and excellent redox properties, CeO_2_ is widely applied in the precision polishing of silicon-based materials like glass.

With the continuous progress of information technology, integrated circuits are evolving towards more interconnect layers and smaller feature sizes. This has led to higher requirements for the global planarization of substrate surfaces [9]. To exercise precise control over the morphology, size, structure, and chemical properties of CeO_2_ abrasives is crucial for the development of integrated circuits. Research has shown that the proper selection of abrasives with an optimal medium size can optimize processing efficiency and surface quality. Spherical abrasives, in particular, have been found to minimize the occurrence of scratches and surface defects [10]. However, common problems such as broad size distribution and agglomeration tendencies in spherical CeO_2_ abrasives have a negative impact on improving the polished surface quality [11]. Additionally, modifying the surface chemical composition of CeO_2_ by increasing the content of Ce^3+^ and oxygen vacancies has been proven to be an effective strategy to enhance CMP activity and material removal efficiency [12,13]. The development of CeO_2_ NPs with suitable particle size, high sphericity, narrow size distribution, and high surface activity is key to overcoming the bottlenecks in CMP processes for advanced integrated circuit manufacturing.

Currently, common synthesis methods for CeO_2_ include hydrothermal/solvothermal synthesis, precipitation, microemulsion, and sol–gel techniques. Each method possesses distinct characteristics but faces varying limitations in morphology control or process complexity. The hydrothermal/solvothermal method generally yields CeO_2_ NPs with relatively uniform morphology. Wang et al. [14] synthesized monodisperse 100 nm nanospheres via the solvothermal method using ethanol and water as solvents, aided by PVP. They proposed that the crystal growth mechanism for spherical nanocrystals involves directed aggregation and Ostwald ripening. Taniguchi et al. [15] obtained spherical CeO_2_ aggregates with a Ce^3+^ concentration as high as 57.4% via a triethylene glycol-assisted solvothermal method. Although hydrothermal methods can yield products with excellent properties, they often rely on complex solvents, expensive reagents, and high-pressure reaction conditions, resulting in high energy consumption and harsh synthesis conditions. The precipitation method, characterized by its simplicity and elimination of washing and purification steps, is widely adopted. Suresh et al. [16] successfully synthesized CeO_2_ NPs using a simple precipitation method with cerium nitrate and ammonia, but the samples exhibited significant agglomeration after the annealing process. Bao et al. [17] introduced hypergravity technology combined with precipitation to achieve highly dispersed, near-spherical CeO_2_ with a diameter of 200 nm. Although this effectively mitigated particle agglomeration, the synthesis of high-performance products relied on hypergravity equipment, which is energy-intensive and involves lengthy reaction cycles. Ren et al. [18] employed liquid-phase precipitation under nitrogen atmosphere to synthesize 26.84 nm near-spherical particles. While nitrogen suppression effectively inhibited agglomeration and controlled morphology, it substantially increased process complexity. Large-scale production also faces challenges in gas uniformity and safety management. He et al. [19] combined microemulsion and homogeneous precipitation methods to produce particles with good monodispersity and narrow size distribution. However, this approach also requires substantial amounts of organic reagents and surfactants, coupled with relatively low yields, making it difficult to meet the demands of green, economical large-scale production. Therefore, while methods exist for synthesizing spherical CeO_2_ NPs at the laboratory scale, achieving a good balance between precise control of particle morphology and size and large-scale production remains challenging. Developing a synthesis strategy that enables efficient control of morphology and size under mild conditions, while also offering process simplicity and scalability potential, is key to advancing high-performance CeO_2_ NPs from the laboratory to practical applications.

The molten salt method is considered a preparation process with significant industrial application potential [20]. The strong polarizing force and rapid mass transfer capability of molten salts can effectively reduce reaction temperatures and shorten reaction times. This method primarily utilizes molten salts as the reaction medium, promoting reactions through ionic polarization, and ultimately obtaining high-purity products by removing salts via water washing. Lan and co-researchers [21,22] established two fundamental synthesis systems—KCl-LiCl and KOH-NaOH—and revealed that atmosphere control is a key strategy for introducing oxygen vacancies and enhancing catalytic performance. Mao et al. [23] optimized the precursor to Ce(NO_3_)_3_·6H_2_O to synthesize smaller nanoparticles, confirming that precursors influence the size and morphology of synthesized nanofibers. Additionally, Ma et al. [24] emphasized that increasing calcination temperature and ion doping helps improve oxygen vacancies on the ceria surface. However, previous literature has primarily focused on salts such as KOH, NaOH, KCl, and LiCl as molten salts. Compared to these salts, the lithium nitrate-potassium nitrate mixture with a molar ratio of 43:57 exhibits a significantly lower melting point of only 132 °C. This property creates favorable conditions for synthesizing CeO_2_ at lower temperatures, offering substantial energy-saving advantages. Therefore, the KNO_3_-LiNO_3_ eutectic molten salt system warrants further attention and investigation. Furthermore, current research on the molten salt method primarily focuses on achieving basic CeO_2_ synthesis or enhancing its catalytic performance through component doping and atmosphere control. In-depth, systematic exploration remains lacking in precisely regulating spherical morphology, size uniformity, and dispersion stability. The particle growth mechanism during CeO_2_ synthesis via the molten salt method also remains unclear and requires further investigation.

Under this background, this work focuses on the controlled synthesis and mechanism analysis of dimensionally uniform spherical CeO_2_ NPs. Specifically, we synthesized spherical CeO_2_ NPs via a one-step calcination process using a KNO_3_-LiNO_3_ eutectic molten salt as the medium. This study systematically investigated the effects of cerium source type, template agent type and dosage, calcination process, and other parameters on the morphology, size, and dispersion of CeO_2_ NPs. It revealed the mechanism by which cerium source anions regulate micelle size, resolving the issue of excessively broad particle size distribution. The study also elucidated the influence of the poly(ethylene oxide)/poly(propylene oxide) (PEO/PPO) segment structure in block copolymers (Pluronic F127/P123) on micelle size. By employing Pluronic copolymers with varying degrees of polymerization, it is feasible to synthesize particles of different sizes, thereby establishing a simple and size-tunable route for preparing spherical CeO_2_ NPs. Building upon this research, the calcination process (temperature, heating rate, soak time) and F127 dosage were optimized to refine particle morphology and size. Through TG-DSC, TG-FTIR, and microstructural characterization, the CeO_2_ synthesis process was thoroughly investigated, revealing its formation mechanism. This provides a new theoretical foundation and reference for achieving controlled synthesis of high-performance CeO_2_ NPs for CMP applications.

## 2. Experimental Procedure

### 2.1. Materials

Cerium(IV) sulfate tetrahydrate (Ce(SO_4_)_2_•4H_2_O), cerium(IV) nitrate (Ce(NO_3_)_4_), cerium(III) nitrate hexahydrate (Ce(NO_3_)_3_•6H_2_O), lithium nitrate (LiNO_3_), Pluronic^®^ F127 (EO_98_PO_67_EO_98_), and P123 (EO_21_PO_67_EO_21_) were of analytical grade and supplied by Macklin Biochemical Technology Co., Ltd. (Shanghai, China). Potassium nitrate (KNO_3_) was purchased from Aladdin Biochemical Technology Co., Ltd. (Shanghai, China). All chemicals were used without further purification. Deionized water was used throughout the experimental process.

### 2.2. Synthesis of CeO_2_ NPs

A mixture of 1 mmol of cerium source (Ce(SO_4_)_2_•4H_2_O, Ce(NO_3_)_4_, or Ce(NO_3_)_3_•6H_2_O), 4.3 mmol LiNO_3_, and 5.7 mmol KNO_3_ was blended with an appropriate amount of F127 or P123. The mixture was then ground thoroughly in an agate mortar until homogeneous. The resultant mixture was transferred to a cylindrical alumina crucible and subjected to a predetermined calcination program in a muffle furnace (e.g., heated to 400 °C at a rate of 6 °C/min and held for 3 h). After the muffle furnace cooled naturally to ambient temperature, the product was collected and repeatedly washed with deionized water via centrifugation. These washing procedures continued until no SO_4_^2−^ was detected in the waste liquid, and this determination was confirmed using a BaCl_2_ solution. The final purified powder was then dried at 60 °C for 8 h to obtain the CeO_2_ sample.

### 2.3. Characterization

The reaction process was analyzed using simultaneous thermogravimetric-differential scanning calorimetry (TGA-DSC, TGA/DSC 1/1600 LF, Zurich, Switzerland) and thermogravimetry–Fourier transform infrared spectroscopy–gas chromatography/mass spectrometry (TG-FTIR, TGA 8000–Spectrum 3–Clarus 690 SQ8T, Waltham, MA, USA). The crystal structure and crystallinity of the samples were characterized by X-ray diffraction (XRD, Rigaku Ultima IV, Tokyo, Japan). The morphology and structural information of the particles were observed using scanning electron microscopy (SEM, Hitachi SU8600, Tokyo, Japan) and transmission electron microscopy (TEM, JEOL JEM-F200, Akishima, Japan). The chemical states of surface elements were analyzed by X-ray photoelectron spectroscopy (XPS, Thermo Scientific K-Alpha, Waltham, MA, USA).

### 2.4. Data Analysis and Statistical Methods

The crystallite size was calculated using the Debye-Scherrer formula based on the full width at half maximum (FWHM) of the (111) diffraction peak in the XRD pattern. The lattice parameters were determined from the peak position according to Bragg’s law of diffraction and the formula. The particle size distribution was obtained by measuring the diameters of over 100 particles from SEM images using Image J software (version 1.53a), and the data are presented as mean ± standard deviation. XPS spectra were fitted and analyzed using the Avantage software (version 6.9.0). The Ce 3d and O 1s spectra were deconvoluted using a Gaussian-Lorentzian function. The proportions of Ce^3+^ and oxygen vacancies were calculated based on the area of the corresponding characteristic peaks after fitting. All XPS data were calibrated prior to fitting by referencing the C 1s peak (binding energy = 284.8 eV).

## 3. Results and Discussion

### 3.1. Thermal Analysis

Thermal analysis is a valuable tool that offers insights into the phase transformation behavior and compositional changes of multi-component systems during calcination. To gain a deep understanding of the thermal decomposition behavior of the mixed system composed of Ce(SO_4_)_2_•4H_2_O, block copolymer F127, and KNO_3_–LiNO_3_ eutectic salt, a combined TG–DSC and TG–FTIR analysis was systematically carried out under an air atmosphere.

**Figure 1:**
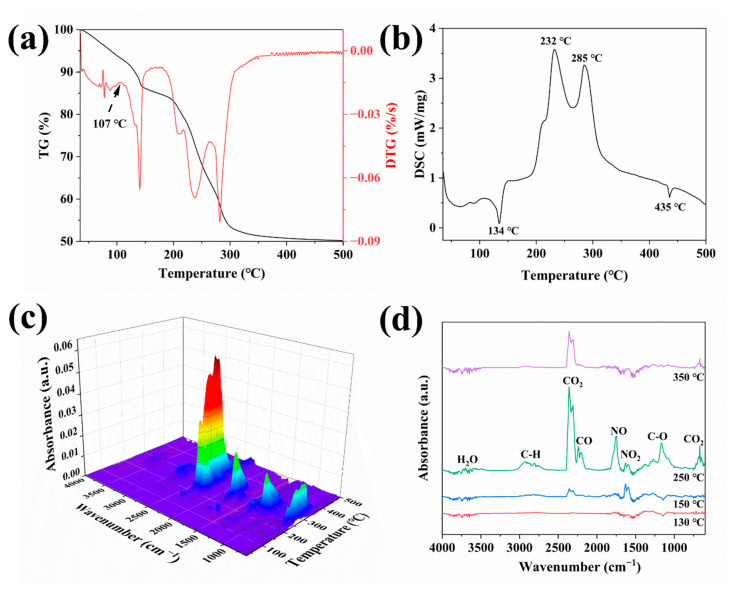
Thermal analysis of the precursor mixture: (**a**) TG-DTG curves; (**b**) DSC curve; (**c**) 3D-FTIR spectrum of gases evolved during heating and (**d**) FTIR spectra at characteristic temperatures.

As shown in Figure 1a, the TG–DSC curves of the mixed system are presented. The TG curve shows that the weight loss during the entire calcination process can be divided into two main stages. The initial mass loss occurs within the temperature range of 30–107 °C, which is due to the release of both physically adsorbed and chemically bound water. This process corresponds to the broad endothermic peak observed near 80 °C on the DSC curve.

The second stage, from 107 to 500 °C, is the primary pyrolysis region. In this region, the mass loss results from the thermal decomposition of the cerium precursor and F127, along with the formation of cerium oxide. Multiple thermal events can be seen in the DSC curve within this stage. A distinct endothermic peak at around 134 °C is consistent with the melting of the LiNO_3_ and KNO_3_ mixed salts. Previous studies have shown that a mixture of lithium nitrate and potassium nitrate at a molar ratio of 43:57 forms a eutectic compound with a melting point of 132 °C [25]. This finding is in line with the present result, suggesting the formation of a molten salt medium at this temperature, which facilitates subsequent ion exchange, cerium salt decomposition, and the synthesis of CeO_2_ NPs.

Two prominent exothermic peaks, at 232 °C and 285 °C, correspond to the thermal oxidative decomposition of the organic component F127. The endothermic effect observed at 436 °C can be attributed to the boiling process of the binary nitrate system.

To clarify the composition of gaseous products released during the thermal decomposition of the cerium precursor, a TG-FTIR analysis was then performed on the mixture. Figure 1c shows the three-dimensional FTIR spectra of the gases evolved during pyrolysis in an air atmosphere. Figure 1d shows the IR absorption spectra of the gases at four characteristic temperatures: 130 °C, 150 °C, 250 °C, and 350 °C.

The IR spectra show a stretching vibration peak of H_2_O between 3500 and 3760 cm^−1^. Absorption peaks corresponding to CO_2_ are observed in the wavenumber regions of 2260–2400 cm^−1^ and 670 cm^−1^ [26]. Peaks located between 2100–2260 cm^−1^ and at 1755 cm^−1^ are assigned to CO and NO, respectively [27]. The absorption peaks at 1627 cm^−1^ and 1588 cm^−1^ are attributed to NO_2_ [28]. Peaks in the regions of 2750–3050 cm^−1^ and 1100–1300 cm^−1^ correspond to the stretching vibrations of C-H and C-O bonds, respectively, originating mainly from organic pyrolysis products like alkanes, alcohols, and phenols [26]. It can be concluded from the gas evolution profiles at different temperatures that the thermal decomposition of F127 mainly produces CO_2_, CO, and light hydrocarbons. In contrast, the cerium precursor calcined in the molten salt mainly releases nitrogen oxides (NO and NO_2_). These results confirm that after the melting of the eutectic nitrate, cerium sulphate first undergoes an ion exchange reaction with NO_3_^−^ to form the intermediate product Ce(NO_3_)_4_, which then decomposes to CeO_2_. This inference is consistent with the experimental detection of SO_4_^2-^ ions in the calcined residue, thus providing strong evidence to support this hypothesis.

Furthermore, within the temperature range of 130–170 °C, the ion-exchanged cerium nitrate begins to decompose slowly, with NO_2_ being the dominant gaseous product. It has been shown that the decomposition rate of the cerium salt increases significantly at temperatures ranging from 200 to 350 °C. Concurrently, NO becomes the main product. Considering the previously described thermal analysis results, the conversion of Ce(SO_4_)_2_•4H_2_O to CeO_2_ during calcination can be described by the following reaction:

1. Dehydration Stage:
  Ce(SO_4_)_2_•4H_2_O → Ce(SO_4_)_2_ + 4H_2_O ↑ (1)
2. Ion Exchange Stage:
  Ce(SO_4_)_2_ + 4NO_3_^−^ → Ce(NO_3_)_4_ + 2SO_4_^2−^ (2)
3. Thermal Decomposition Stage:
  Ce(NO_3_)_4_ → CeO_2_ + 4NO ↑ + 3O_2_ ↑ (3)
  Ce(NO_3_)_4_ → CeO_2_ + 4NO_2_ ↑ + O_2_ ↑ (4)

### 3.2. Effect of Reaction Conditions on CeO_2_ NPs

#### 3.2.1. Influence of Cerium Salt and Surfactant Type

**Figure 2:**
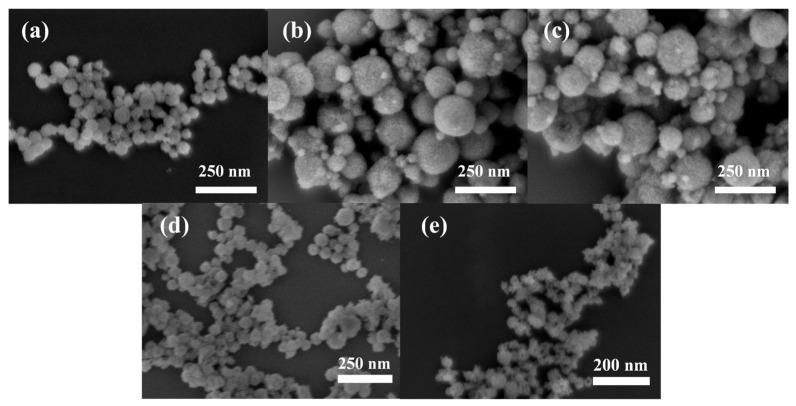
SEM images of CeO_2_ NPs prepared using different cerium sources and surfactants: (**a**) Ce(SO_4_)_2_•4H_2_O; (**b**) Ce(NO_3_)_4_; (**c**) Ce(NO_3_)_3_•6H_2_O; (**d**) F127; (**e**) P123.

The choice of cerium precursor has a significant impact on the morphology and chemical properties of the resultant CeO_2_. Therefore, to screen for suitable precursors, three different cerium sources (Ce(SO_4_)_2_·4H_2_O, Ce(NO_3_)_4_, and Ce(NO_3_)_3_·6H_2_O) were selected. The precursor addition amount was uniformly controlled at 1 mmol, and experiments were conducted using 0.4 g of F127 as the template. The morphologies of the obtained samples were characterized by SEM, as shown in Figure 2a–c.

The results reveal that CeO_2_ prepared from Ce(SO_4_)_2_•4H_2_O consists of uniform nanospheres with an average particle size of 50 nm. In contrast, products derived from Ce(NO_3_)_4_ or Ce(NO_3_)_3_•6H_2_O show a bimodal size distribution composed of spherical particles of approximately 30 nm and 150 nm, leading to poor overall uniformity. This difference in morphology indicates that SO_4_^2−^ plays a crucial role in regulating the size uniformity of CeO_2_ NPs within the molten salt medium. It is hypothesized that divalent sulfate ions adjust micelle dimensions, thus inhibiting the formation of oversized particles. Therefore, Ce(SO_4_)_2_•4H_2_O is more suitable as a precursor for synthesizing CeO_2_ NPs with uniform particle size and was selected as the base precursor for subsequent optimization.

To further investigate the effect of block copolymer type on product morphology, CeO_2_ was synthesized using Ce(SO_4_)_2_•4H_2_O as the cerium source with two block copolymers, F127 and P123, respectively. The copolymer loading was uniformly set at 0.4 g in all experiments. SEM images of the synthesized CeO_2_ are shown in Figure 2d,e.

The results show that both F127 and P123 can direct the formation of a spherical morphology. However, the sample prepared with P123 has a smaller particle size, approximately 40 nm in diameter. This observation can be attributed to the molecular structures of the two copolymers. Despite their structural similarity, characterized by the presence of PEO and PPO blocks with equivalent degrees of polymerization of PPO, significant differences exist in the lengths of their PEO blocks, with degrees of polymerization of 98 and 21, respectively. Previous studies have shown that among Pluronic—type block copolymers with similar PPO block lengths, those with longer PEO segments form micelles with smaller aggregation numbers and larger sizes [29]. During the synthesis of CeO_2_ NPs, the initial nanoparticles undergo directed assembly and confined growth templated by spherical micelles; thus, the micelle size directly affects the final particle size. This result also demonstrates that using block copolymers with different degrees of polymerization as templates enables control over particle size, thereby providing a simple and size-tunable route for preparing spherical CeO_2_ NPs. Based on the above results, in order to balance the morphology and moderate particle size of the product, F127 was selected for the optimization of the subsequent process.

#### 3.2.2. Influence of Calcination Process

In the molten salt synthesis of CeO_2_ NPs, the calcination conditions, namely temperature, heating rate, and holding time, play a crucial role in regulating the dissolution of nitrates, the decomposition of the precursor and block copolymer F127, as well as the rates of crystal nucleation and growth. Thus, the parameters of calcination temperature, heating rate, and holding time were systematically varied to analyze their individual effects on the resulting CeO_2_ product.

**Figure 3:**
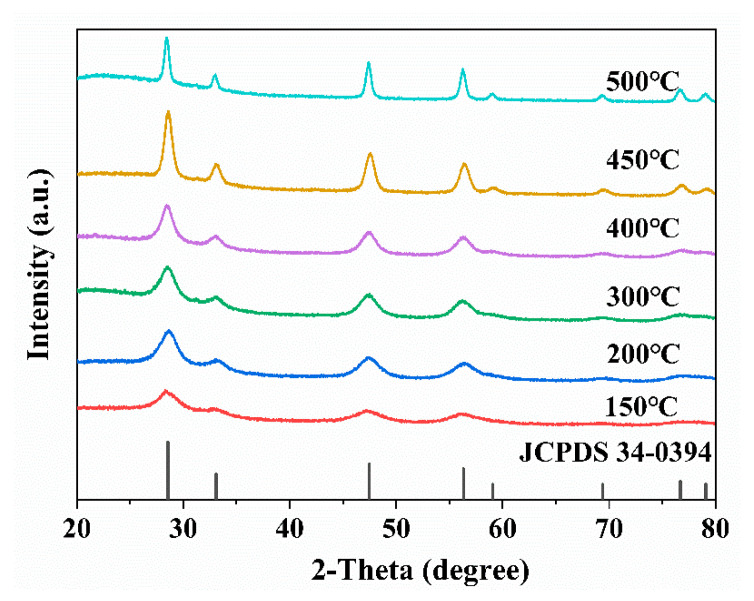
XRD patterns of CeO_2_ synthesized at different calcination temperatures.

Under fixed experimental parameters, CeO_2_ NPs were synthesized by systematically varying the calcination temperature (150, 200, 300, 400, 450, and 500 °C). As shown in Figure 3, the XRD patterns of CeO_2_ samples prepared at different temperatures are presented. All diffraction peaks showed a high degree of compatibility with the standard card of CeO_2_ (JCPDS No. 34-0394), corresponding to a face—centered cubic structure (space group Fm3m). No impurity phases were detected. The prominent diffraction peaks are located at 2θ = 28.5 °, 33.1 °, 47.5 °, 56.3 °, 59.1 °, 69.4 °, 76.7 °, and 79.1 °, which are assigned to the (111), (200), (220), (311), (222), (400), (331), and (420) crystal planes, respectively. It is notable that phase—pure CeO_2_ can be successfully synthesized even at a temperature as low as 150 °C. This phenomenon can be mainly attributed to the highly polar solvent environment provided by the molten nitrate salt, which effectively disrupts the chemical bonds between metal ions [30].

The liquid medium formed by the molten salt has also been shown to significantly enhance ion mobility and promote reactant mass transfer, thus substantially reducing the synthesis temperature. However, the sample synthesized at lower temperatures exhibits broadened diffraction peaks, indicating relatively poor crystallinity. As the calcination temperature increases, the diffraction peak intensity intensifies and the FWHM narrows, indicating enhanced crystallinity and larger particle size. This result aligns with findings reported by Kravtsov et al. [31]. However, at 500 °C, the diffraction peak intensity decreases compared to 450 °C, potentially due to the collapse of the porous nanocluster structure at this temperature. The structural collapse led to denser particles and a significant reduction in the material’s specific surface area, which in turn caused the observed decrease in CeO_2_ diffraction peak intensity.

According to the Debye–Scherrer equation, crystallite size calculation depends on the FWHM of the diffraction peak and the Bragg angle [32]. The lattice parameters and crystallite sizes (D), calculated using the (111) plane date, are summarized in Table 1. It can be observed that as the calcination temperature increases, the crystallite size of CeO_2_ gradually enlarges. This result is consistent with that reported by Aminzare et al. [33], indicating that calcination temperature directly influences the particle characteristics of the final powder. Specifically, when the calcination temperature rises from 150 °C to 400 °C, the grain size increases only slightly. However, when the temperature was increased to 500 °C, a marked increase in crystallite size was observed, from approximately 5.3 nm to 13.2 nm. This finding suggests that elevated temperatures significantly promote crystallite growth, which is consistent with classical nucleation and growth theory. Typically, cerium oxide crystallite formation involves both nucleation and growth processes [34]. The balance between nucleation and crystal growth is crucial in determining the final crystallite size [35]. In the range of lower temperatures (150–400 °C), the nucleation rate has a predominant influence, leading to the formation of a large number of minute nuclei. Conversely, at elevated calcination temperatures (450–500 °C), the crystal growth rate exceeds the nucleation rate, resulting in a substantial increase in crystallite size.

**Figure 4:**
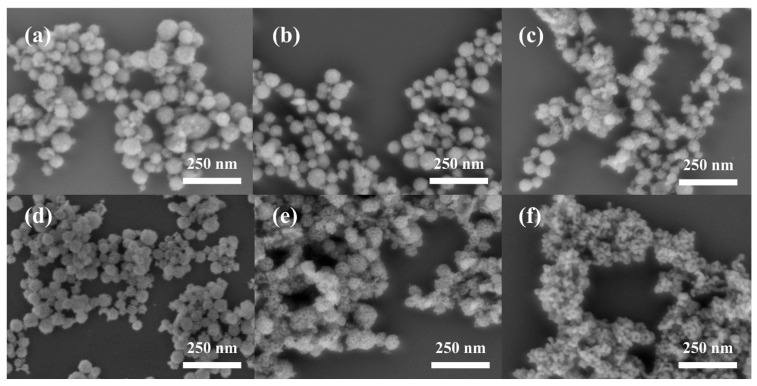
SEM images of CeO_2_ NPs synthesized at different temperatures: (**a**) 150 °C; (**b**) 200 °C; (**c**) 300 °C; (**d**) 400 °C; (**e**) 450 °C; (**f**) 500 °C.

**Figure 5:**
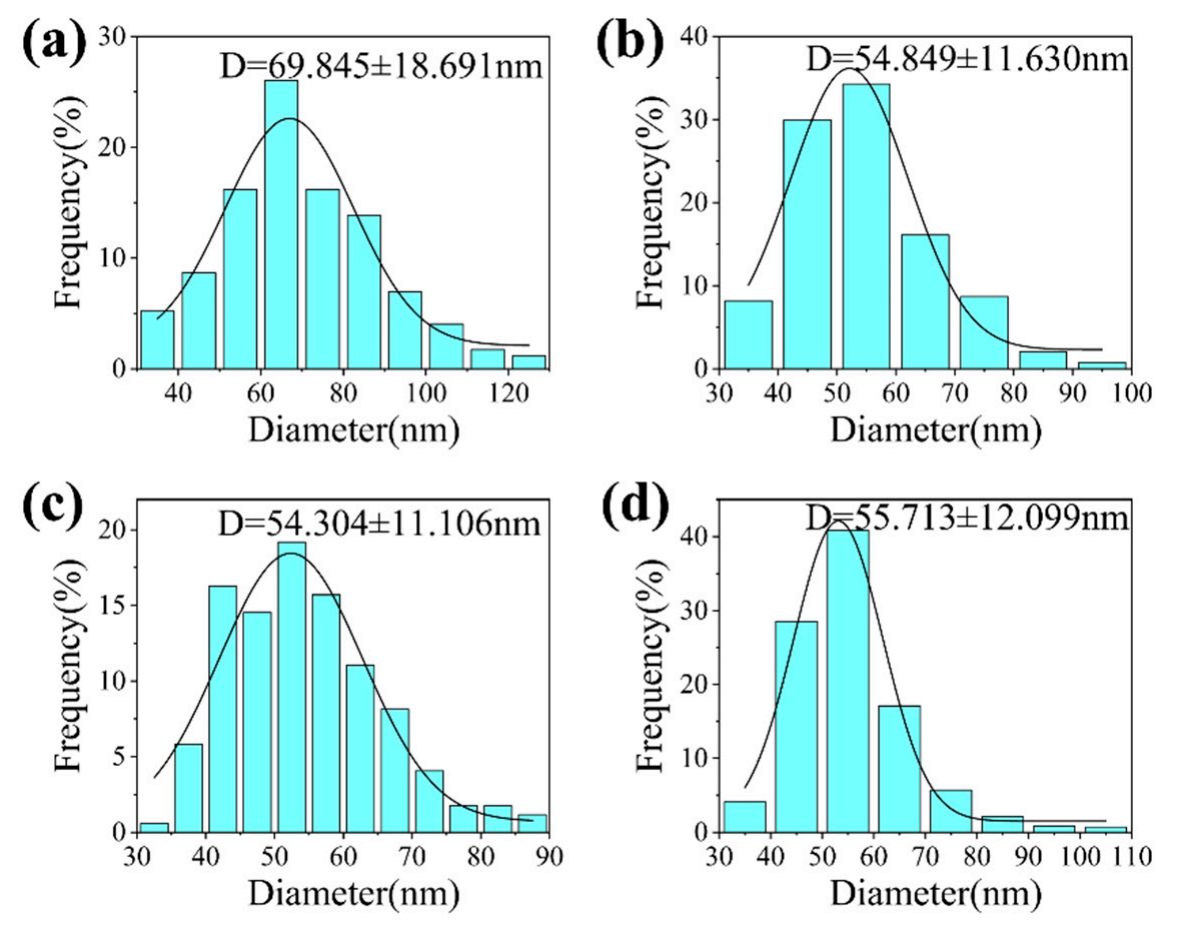
Particle size distribution histograms of CeO_2_ NPs synthesized at different temperatures: (**a**) 150 °C; (**b**) 200 °C; (**c**) 300 °C; (**d**) 400 °C.

**Figure 6:**
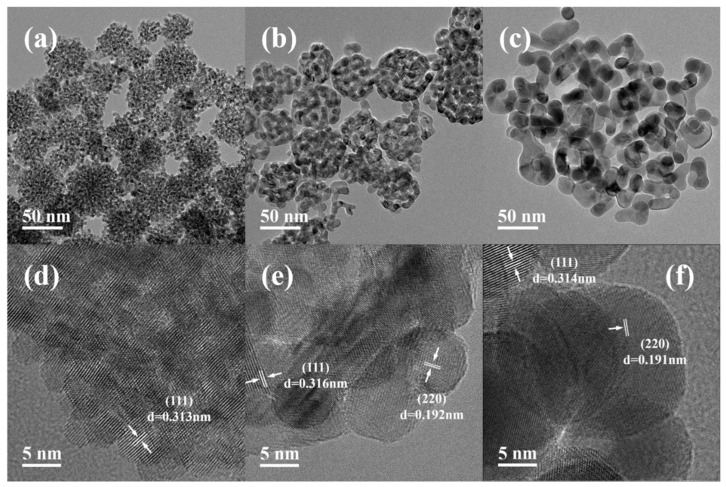
TEM and HRTEM images of CeO_2_ NPs synthesized at different temperatures: (**a**,**d**) 400 °C; (**b**,**e**) 450 °C; (**c**,**f**) 500 °C.

Figure 4 and Figure 5 show the SEM images and the particle size distribution diagrams of CeO_2_ synthesized at different reaction temperatures. The CeO_2_ obtained at 150 °C exhibited a well—defined spherical morphology with an average particle size of approximately 70 nm; however, the size distribution was broad and the uniformity was relatively poor. As the calcination temperature was elevated to the range of 200–400 °C, there was a slight decrease in the average particle size, which stabilized at approximately 55 nm, accompanied by a narrower size distribution. Overall, variations in calcination temperature within this range did not significantly affect particle morphology and size. This trend differs from the findings reported by Lan et al. [22]. Their study indicated that when employing the molten salt method, the average particle size of CeO_2_ increased substantially from 30 nm to 240 nm as the calcination temperature rose from 200 °C to 500 °C. The primary reason for this discrepancy lies in differing morphology control mechanisms. In this experimental system, the surfactant F127 exerts a dominant regulatory effect on particle morphology and size. At a temperature of 450 °C, it is clearly observable that the particles have rough surfaces and a porous structure. Furthermore, the morphology and microstructure of the products synthesized at 400 °C, 450 °C, and 500 °C were further characterized by TEM and HRTEM, as shown in Figure 6.

As shown in Figure 6a,b, the CeO_2_ obtained at 400 °C and 450 °C shows a spherical morphology with a porous structure and an average particle size of approximately 50 nm, thus corroborating the SEM observations. Figure 6d,e further reveal that the CeO_2_ synthesized under these conditions consists of spherical aggregates approximately 50 nm in size, which are composed of finer crystallites measuring 5 nm and 9 nm, respectively. The formation of such aggregates can be attributed to the high surface energy and specific surface area of the cerium nanocrystallites, which drive the primary nanoparticles to assemble into larger aggregates to minimize the total energy of the system [36]. When the same crystal faces come into contact, the surface energy is lower; therefore, the aggregation of nanocrystals must be oriented [32]. However, misalignment may occur at the interfaces between initially attached nanocrystals under practical conditions, resulting in regions of imperfect alignment and leading to the presence of dislocations and defects [37]. The HRTEM images show lattice fringes that confirm the high crystallinity of the CeO_2_, with an interplanar spacing of 0.3 nm corresponding to the (111) plane of the cubic fluorite phase of CeO_2_.

Compared to spherical clusters formed at 450 °C, those formed at 400 °C exhibit smaller intergranular spacing within the lattice. When the calcination temperature was increased to 500 °C, the synthesized CeO_2_ transitioned toward a single-crystal structure. Its morphology featured not only dispersed spherical particles measuring 15–20 nm in size but also “V”-shaped and “U”-shaped structures. The morphological transition occurring at 500 °C also corresponds to the anomalous decrease in XRD peak intensity. These morphological and structural evolutions primarily stem from the profound influence of calcination temperature on the decomposition behavior of the template agent and the particle growth mechanism, leading to the imbalance of multiple kinetic processes. In this synthesis system, surfactant F127 serves as a soft template. The metal precursor binds to it, forming an intermediate hybrid structure [38]. During thermal treatment, the template is progressively removed, ultimately yielding a porous product. However, this approach presents challenges, such as the collapse of ordered structures during template removal [39]. Furthermore, block copolymers like F127 exhibit limited thermal stability, decomposing rapidly even under inert conditions. When calcination temperatures exceed 400 °C, maintaining the structural integrity of mesoporous metal oxides under high temperatures becomes a significant challenge [40]. Therefore, it can be clearly observed that as the calcination temperature gradually increases to 450 °C and 500 °C, the collapse of nanoclusters becomes progressively more severe. At 500 °C, severely agglomerated single-crystal particles formed instead of porous nanoclusters. These phenomena can be attributed to the accelerated decomposition rate of F127 at higher temperatures, causing premature collapse of the spherical micelle template structure. This failure prevents effective guidance for primary particles to assemble orderly along the micelle-directed structure. On the other hand, at the higher temperature of 500 °C, some spatially adjacent spherical particles still undergo oriented adhesion, aligning along the same crystallographic direction to reduce the system’s total energy. Moreover, higher calcination temperatures promote the fusion and sintering of adjacent particles into single crystals, yielding “V”-shaped, “U”-shaped, or elongated CeO_2_ structures [41]. Therefore, to simultaneously ensure the spherical morphology of CeO_2_, a narrow particle size distribution, good crystallinity, and complete removal of the template agent, the optimal calcination temperature was determined to be 400 °C.

Based on the determined calcination temperature, the effects of heating rate and soaking time on the morphology and size distribution of CeO_2_ particles were further investigated.

I. Effects of Heating Rate

**Figure 7:**
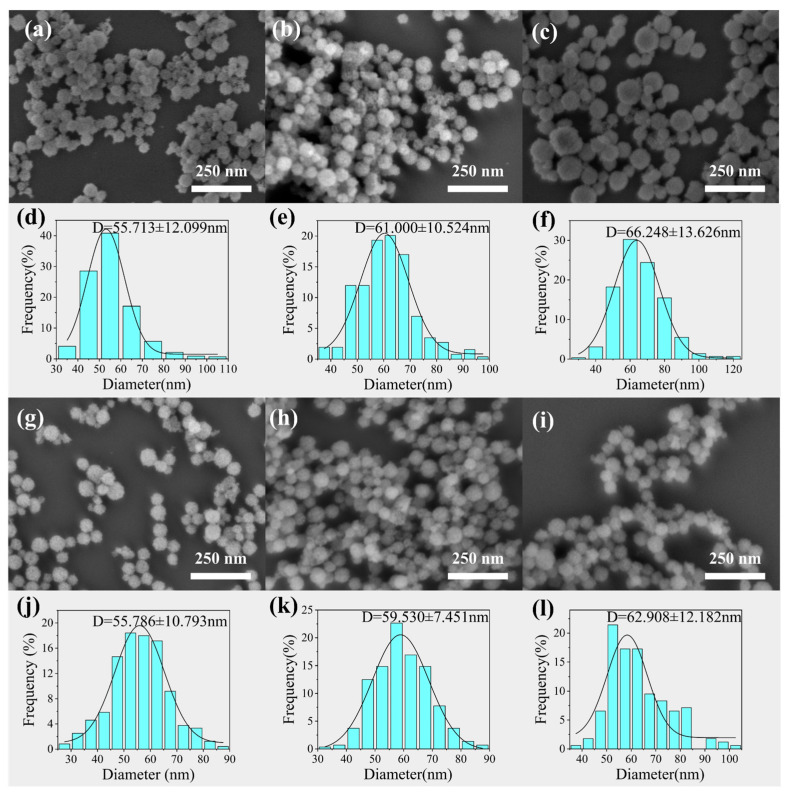
SEM images and particle size distribution histograms of CeO_2_ NPs synthesized at different heating rates and holding times: (**a**,**d**) 2 °C/min; (**b**,**e**) 6 °C/min; (**c**,**f**) 10 °C/min; (**g**,**j**) 30 min; (**h**,**k**) 3 h; (**i**,**l**) 6 h.

Figure 7a–f presents the SEM images and corresponding particle size distribution histograms of samples synthesized at different heating rates (2, 6, and 10 °C/min). The results show that, under the same calcination temperatures, the average particle size of CeO_2_ increased slightly with the increase in heating rate.

- At a heating rate of 2 °C/min, a part of extremely fine particles existed in the product.
- At a rate of 10 °C/min, a small number of large particles with dimensions greater than 100 nm were found.
- The optimal heating rate was 6 °C/min. Under this rate, the proportion of undersized particles decreased, resulting in a uniform particle size with a narrow distribution.

This phenomenon can be attributed to the fact that at higher heating rates, after nitrate melting, the system temperature reached during the micellization of F127 was higher. It has been shown that the aggregation number of block copolymer micelles increases with temperature, leading to a moderate increase in micellar size [42,43]. Therefore, higher heating rates promote more F127 molecules to be incorporated into individual micelles, thereby reducing the total number of micelles while increasing their size. This leads to a higher cerium ion loading per micelle and ultimately larger CeO_2_ NPs. Moreover, excessively high heating rates may cause temperature inhomogeneities within the system during heating, resulting in broader particle size distributions in the final product. Therefore, 6 °C/min is the optimal heating rate.

II. Effects of Soaking Time

Subsequently, the effects of holding time (0.5, 3, 6 h) were investigated under calcination conditions of 400 °C and a heating rate of 6 °C/min. As shown by the experimental results in Figure 7g–l, the average particle size of the spherical CeO_2_ particles did not change significantly under different holding times. This indicates that under the selected calcination temperature and heating rate, the particle size gradually stabilizes and remains nearly constant once the holding time exceeds a certain threshold.

- However, when the holding time was extended to 30 min, a large number of fine particles in the range of 10–20 nm were observed in the product.
- When the holding time was extended to 6 h, abnormal growth of some particles occurred, resulting in some oversized particles with diameters greater than 100 nm.

This phenomenon can be explained by the Ostwald ripening mechanism. As the holding time increased, the proportion of fine particles gradually decreased, and the particle size distribution became significantly narrower, indicating enhanced homogeneity. The Ostwald ripening mechanism can explain the phenomenon under discussion. In conditions of elevated temperatures, particles with diameters smaller than the critical radius are prone to dissolution due to their higher surface energy. Then, the diffusion of dissolved species through the molten salt medium and redeposition onto the surfaces of larger particles promote particle size uniformity [44]. Therefore, appropriately extending the holding time facilitates the diffusion and rearrangement processes within the system, thereby improving the monodispersity of the products. After comprehensive consideration of the morphology, size distribution, control, and energy efficiency, the optimal holding time was determined to be 3 h. Under these conditions, effective particle size homogenization can be achieved while avoiding abnormal particle growth caused by overly long holding times.

III. Optimal Calcination Parameters

Therefore, after comprehensively considering product morphology, particle size control, and size distribution uniformity, the optimal calcination parameters were determined as follows: a calcination temperature of 400 °C, a heating rate of 6 °C/min, and a holding time of 3 h. Under these conditions, the synthesis resulted in CeO_2_ NPs with good dispersibility and a uniform size distribution, along with the complete removal of the block copolymer. Subsequent studies were also conducted under these optimal calcination process conditions.

#### 3.2.3. Influence of F127 Dosage

**Figure 8:**
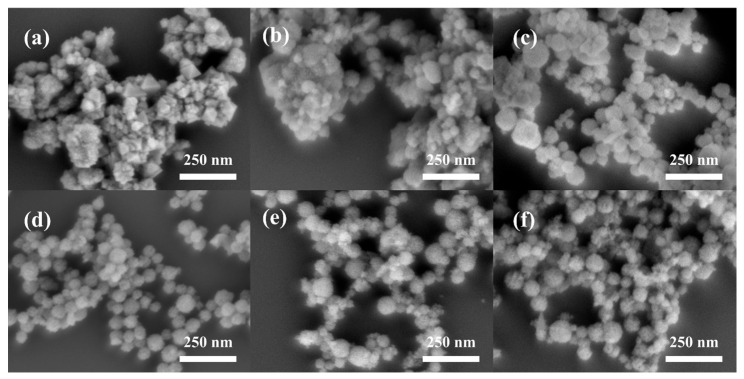
SEM images of CeO_2_ NPs synthesized with different F127 dosages: (**a**) 0 g; (**b**) 0.1 g; (**c**) 0.23 g; (**d**) 0.4 g; (**e**) 0.55 g; (**f**) 0.7 g.

The block copolymer F127 acts as a soft template, playing a crucial role in regulating the spherical morphology and dispersibility of CeO_2_ NPs. In polar solutions, at the critical micelle concentration (CMC), F127 forms micelles, which provide morphological guidance for the growth of CeO_2_, thus influencing the final particle morphology. To determine the optimal amount of template agent, we systematically investigated the influence mechanism of F127 dosage on the morphology, size, and dispersion state of CeO_2_. Under optimal calcination conditions, experiments were conducted with six F127 addition levels: 0, 0.1, 0.25, 0.4, 0.55, and 0.7 g. As shown in Figure 8, without F127, CeO_2_ exhibited an octahedral morphology and block-like structures with severe agglomeration. When 0.1 g of F127 was added, a small number of spherical particles started to appear, but irregular agglomerates still dominated, indicating that this dosage was not enough to direct the formation of a fully spherical nanoscale morphology. As the F127 amount increased to 0.25 g, the irregular block—like structures mostly disappeared, changing to a spherical morphology. However, the particle size distribution remained wide, ranging from tens to hundreds of nanometers, and the dispersibility was still not satisfactory. This implies that F127 began to play a morphological directing role, yet its concentration was insufficient to effectively inhibit particle agglomeration. When the F127 dosage was further increased to 0.4 g, the CeO_2_ NPs showed a well—defined spherical morphology, a significantly narrower size distribution, and much improved dispersibility. At this level, the amount of F127 was sufficient to effectively guide the uniform nucleation and growth of CeO_2_. Increasing the F127 amount further to 0.55 g and 0.7 g led to the appearance of a large number of tiny CeO_2_ NPs. This can be ascribed to the formation of an excessive number of micelles due to the excess F127. This causes a reduction in the ion load per micelle, ultimately producing smaller CeO_2_ NPs. Based on the comprehensive optimization of morphological regularity, size uniformity, and dispersibility, the optimal dosage of F127 was determined to be 0.4 g.

### 3.3. Characterization of Spherical CeO_2_ NPs Synthesized Under Optimal Conditions

Based on the systematic investigation of reaction conditions as described above, the morphology, size, and dispersion of the synthesized CeO_2_ NPs are jointly controlled by selecting the cerium source and template agent, optimizing the calcination parameters, and adjusting the F127 dosage.

By precisely regulating these factors, well-dispersed, spherical CeO_2_ NPs with uniform particle size and distinct morphology were successfully prepared through a one-step calcination process. The optimized synthesis conditions are as follows: Ce(SO_4_)_2_·4H_2_O was used as the cerium source; 0.4 g of F127 was used as the templating agent; and a calcination program with a heating rate of 6 °C/min to 400 °C was employed, followed by a 3 h holding time.

**Figure 9:**
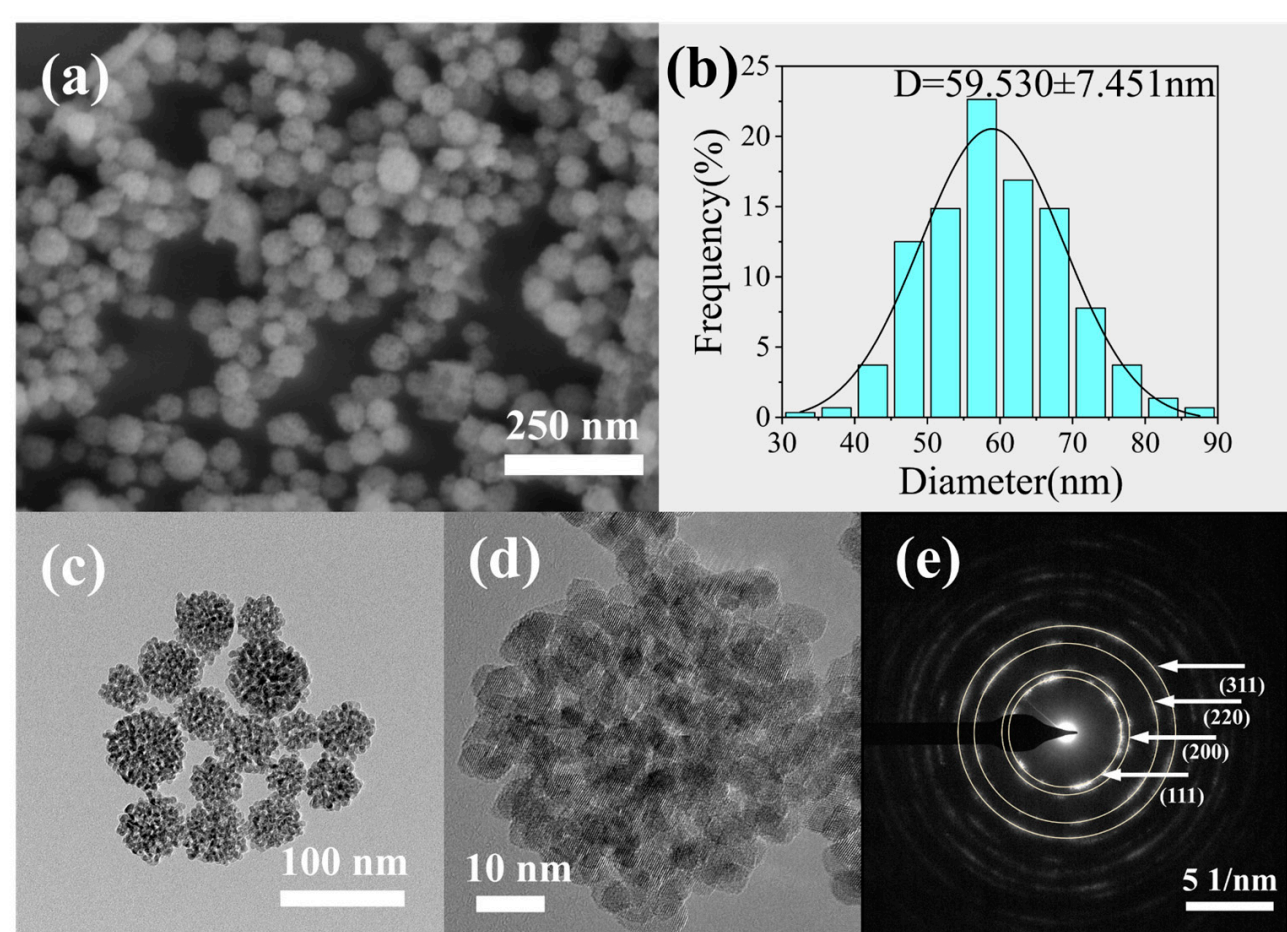
SEM and TEM characterization of CeO_2_ NPs synthesized under optimal conditions: (**a**) SEM image; (**b**) particle size distribution histogram; (**c**) TEM image; (**d**) HRTEM image of a single nanocluster and (**e**) SAED pattern.

Samples characterized by SEM and TEM were prepared under optimal conditions. The results are presented in Figure 9. Figure 9a shows that CeO_2_ exhibits a uniform spherical morphology, with good dispersion and an average particle size of about 60 nm. Figure 9d shows a high-resolution transmission electron microscopy (HRTEM image, which reveals that these microspheres are porous aggregates formed by the oriented stacking of primary nanocrystals measuring 5–7 nm in diameter. These aggregates exhibit relatively high surface roughness. The selected area electron diffraction (SAED) pattern in Figure 9e reveals that each diffraction ring comprises multiple elongated spots. This indicates that the nanospheres consist of numerous small nanocrystals and exhibit polycrystalline characteristics. The diffraction rings, from the innermost to the outermost, correspond sequentially to the (111), (200), (220), and (311) crystal planes of the face-centered cubic CeO_2_ structure.

**Figure 10:**
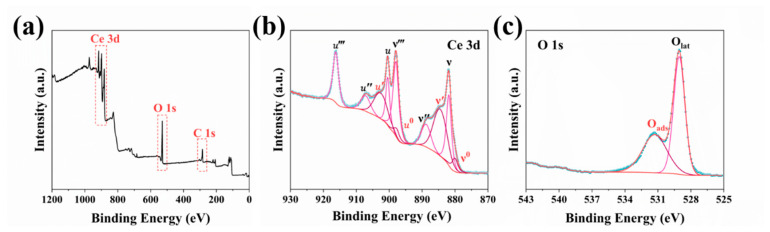
XPS spectra of spherical CeO_2_ NPs synthesized under optimal conditions: (**a**) survey scan; (**b**) Ce 3d; (**c**) O 1s.

Figure 10 presents the results of a comprehensive analysis of the surface elemental composition and chemical states of the samples via X-ray photoelectron spectroscopy (XPS). The survey spectrum (Figure 10a) verifies the presence of Ce and O, along with trace amounts of C, indicating high product purity. This finding aligns with the TEM results. High-resolution spectra of the Ce 3d and O 1s regions were fitted using a combination of Gaussian-Lorentzian function combination.

Figure 10b shows that the high-resolution Ce 3d spectrum was deconvoluted into multiple peaks, revealing the coexistence of Ce^3+^ and Ce^4+^ species. As per previous reports, the spin–orbit splitting peaks of the Ce 3d orbital, specifically the 3d_3/2_ and 3d_5/2_ components, are conventionally denoted as u and ν, respectively.

According to previous reports, the spin–orbit splitting of the Ce 3d orbital generates the 3d_3/2_ and 3d_5/2_ components, which are typically denoted as u and ν, respectively [45]. Specifically, the u, u″, u‴, ν, ν″, and ν‴ peaks are characteristic of Ce^4+^, while the u_0_, u′, ν_0_, and ν′ peaks are assigned to Ce^3+^. The relative concentration of Ce^3+^ on the material surface was quantitatively determined using the following formula [12]: Ce^3+^/(Ce^3+^+Ce^4+^) = area(u_0_, u′, ν_0_, ν′)/total area. The calculated Ce^3+^ content was 40.22%, significantly higher than that in conventional CeO_2_ samples. This demonstrates the effective introduction of substantial Ce^3+^ defects through this synthetic route. Moreover, the presence of Ce^3+^ induces charge imbalance, thus promoting the formation of oxygen vacancies. Therefore, the concentration of Ce^3+^ is closely related to the abundance of oxygen vacancies in CeO_2_.

The fitting results of the high-resolution O 1s spectrum are shown in Figure 10c. Peaks located at binding energies of 531.4 eV and 529.1 eV are assigned to surface-adsorbed oxygen (O_ads_) and lattice oxygen (O_lat_), respectively. These adsorbed oxygen species can interact with defect sites to form oxygen vacancies [46]. The fitting results reveal that the proportion of O_ads_ is 41.18%, further confirming the presence of abundant oxygen vacancy defects in the material. This phenomenon is due to the consumption of CeO_2_ lattice oxygen during the high-temperature decomposition of organic species, resulting in a high concentration of oxygen vacancy defects.

In conclusion, through systematic optimization of multiple parameters, highly spherical nano-CeO_2_ with a narrow size distribution and a well-defined defect structure rich in Ce^3+^ ions and oxygen vacancies were successfully synthesized. The as-prepared material shows promising potential for applications in CMP, especially in the high-precision polishing of silica-based substrates, where it is expected to exhibit high chemical activity and excellent polishing performance.

To comprehensively evaluate the characteristics of CeO_2_ NPs prepared under optimal conditions, the samples from this work were compared with various ceria materials reported in the literature. The statistical results are shown in Table 2. The spherical CeO_2_ NPs prepared in this work exhibit an average particle size of 59.53 ± 7.41 nm. Compared to ceria reported in other studies, it exhibits a narrower size distribution and superior size uniformity. This advantage stems from the micelle templating effect of surfactant F127 and optimized process parameters. Regarding surface chemistry, the Ce^3+^ content in the prepared material reached 40.22%, higher than that reported in other studies. The O_ads_ content also reached a high level of 41.18%. Overall, multi-parameter optimization enabled the preparation of CeO_2_ NPs with high sphericity, narrow size distribution, and rich Ce^3+^/oxygen vacancy content. Abrasives with moderate particle size and good size uniformity are more conducive to achieving smooth, low-damage polished surfaces in CMP. Highly chemically active abrasives enhance chemical interactions with materials like SiO_2_, significantly boosting polishing activity and material removal rates. Consequently, this material holds significant application potential in CMP polishing, particularly demonstrating high reactivity and excellent polishing capability in high-precision silicon dioxide substrate polishing.

### 3.4. Formation Mechanism of Spherical CeO_2_ NPs

**Figure 11:**
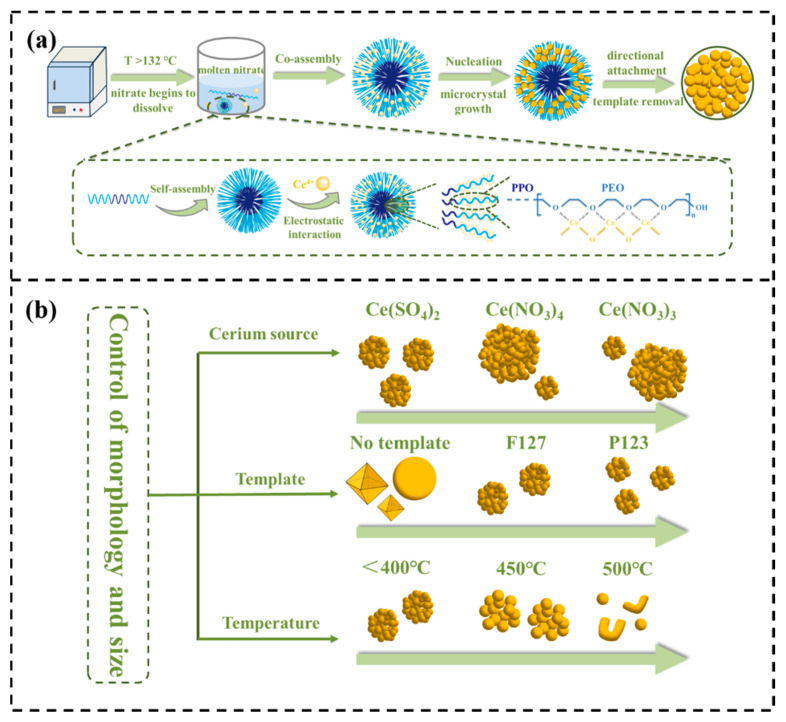
(**a**) Formation mechanism of spherical CeO_2_ NPs and (**b**) schematic illustration of the regulation of particle morphology and size.

Based on the above findings, the proposed mechanism for the formation of spherical CeO_2_ NPs is illustrated in Figure 11. Initial Heating Stage (below 134 °C, the eutectic point of the nitrate salt), the mixture of Ce(SO_4_)_2_·4H_2_O, F127 and nitrate salts is solid initially. The components interact mainly through physical contact. As the temperature rises above 134 °C, the eutectic nitrate salt melts and turns into an ionic liquid phase. It gradually penetrates the space between the F127 and the cerium source. In the molten state, the salt dissociates into anions and cations, showing high polarity, strong ionic capacity, and excellent ion migration properties [50]. In this polar ionic environment, once the concentration of the block copolymer F127 reaches the CMC, its molecules spontaneously self—assemble into spherical micelles. These micelles are then uniformly dispersed in the melt. The hydrophilic PEO segments of F127 extend outward to form the micelle corona, while the hydrophobic PPO blocks curl inward to form the core [51]. Certainly, the PEO segments in F127 can form electrostatic coordination interactions with Ce^4+^ metal ions, leading to crown-ether-type complexes [52]. This forms an organic-inorganic composite precursor unit with micelles as templates and cerium ions are enriched at the interface. This micelle-directed assembly is crucial for the formation of spherical polycrystalline CeO_2_ NPs.

The formation of polycrystalline CeO_2_ NPs consists of two stages: nucleation and growth of primary crystallites, followed by micelle-templated oriented attachment and self-assembly. When the temperature reaches around 150 °C, the cerium precursor starts to decompose thermally, continuously precipitating tiny CeO_2_ crystalline nuclei at the micelle interface. The spherical micelles then act as structural directors for the ordered growth of the CeO_2_ nanocrystals. As the calcination process continues, the pre-formed primary particles experience brief growth. Adjacent nanocrystals then orientally attach under the guidance of F127 micelles, accompanied by crystal plane reconstruction, gradually assembling into uniformly sized, regularly shaped secondary spherical aggregates. During this process, the templating agent decomposes gradually. The final size of the spherical aggregates is closely related to the dimensions of the templating micelles. This relationship reasonably explains the significant differences in diameter observed in spherical CeO_2_ NPs produced using block copolymers with different PEO chain lengths, like F127 and P123.

It should be noted that the calcination temperature plays a vital role in this assembly process. When the calcination temperature is below 400 °C, the decomposition rate of F127 and the assembly rate of CeO_2_ crystallites are balanced, enabling the micellar templates to remain stable until the assembly process is complete, which mainly facilitates the formation of a porous spherical structure. On the contrary, at excessively high temperatures (e.g., 500 °C), the rapid decomposition of F127 causes the micellar structure to collapse prematurely. Without the guidance of the templates, the primary nanocrystals cannot form nanocluster structures. Some spherical particles undergo oriented attachment and sintering, resulting in a final product dominated by severely agglomerated spherical or curved rod-like structures.

## 4. Conclusions

This study employed a molten salt method to precisely control and prepare spherical CeO_2_ NPs through a single-step calcination process. It was found that the type of cerium source anion, surfactant selection, and calcination temperature are critical factors in controlling the morphology and size of the product. The SO_4_^2−^ ions provided by Ce(SO_4_)_2_•4H_2_O suppress the formation of bimodal particle size distributions within the molten salt. Selecting Pluronic surfactants with varying degrees of polymerization (e.g., F127 and P123) enables straightforward control over particle size. Excessively high calcination temperatures (500 °C) can lead to uncontrolled particle morphology. Under optimal conditions, uniform spherical CeO_2_ NPs (59.53 ± 7.41 nm) were synthesized. The particles formed by the aggregation of primary nanocrystals measuring about 5–7 nm, exhibiting surface Ce^3+^ and O_ads_ contents as high as 40.22% and 41.18%. In the field of CMP, their moderate particle size and uniform morphology contribute to reduced surface roughness, while high chemical activity is expected to significantly enhance polishing rates, meeting the high-precision polishing demands of advanced integrated circuits. However, this study also has some limitations. The large volume of wastewater generated after washing exhibits complex composition, containing both KNO_3_ and LiNO_3_ while also introducing SO_4_^2−^ ions derived from precursors. Therefore, effectively removing SO_4_^2−^ from wastewater while simultaneously achieving efficient recovery and recycling of nitrates is essential for green and economic production.

## Figures and Tables

**Table 1:** Lattice parameters and crystallite sizes of CeO_2_ NPs calculated based on the (111) plane.

Temperature/°C	FWHM(111)/deg	d(111)/Å	A/Å	D/nm
500	0.62	3.134	5.428	13.219
450	0.94	3.121	5.406	8.721
400	1.54	3.132	5.425	5.322
300	2.02	3.132	5.425	4.058
200	2.22	3.114	5.394	3.693
150	2.94	3.147	5.451	2.787

**Table 2:** Particle size and surface chemical state of CeO_2_ samples.

Samples	Particle Size/nm	Ce^3+^ content/%	O_ads_ Content/%	Refs.
Single-crystal CeO_2_	40 ± 5	22.03	24.02	[18]
CeO_2_ NPs	59 ± 7	40.22	41.18	This work
Single-crystal CeO_2_	60 ± 12	29.37	30.73	[18]
Porous CeO_2_	79 ± 9	10.74	76.67	[47]
Porous CeO_2_	105	25.37	\	[48]
mCeO_2_	180	35.50	\	[49]
mCeO_2_	200–300	33.88	21.17	[11]

## Data Availability

The original contributions presented in this study are included in the article. Further inquiries can be directed to the corresponding author.
